# Highly Porous Polymer-Derived Bioceramics Based on a Complex Hardystonite Solid Solution

**DOI:** 10.3390/ma12233970

**Published:** 2019-11-30

**Authors:** Hamada Elsayed, Michele Secco, Federico Zorzi, Katharina Schuhladen, Rainer Detsch, Aldo R. Boccaccini, Enrico Bernardo

**Affiliations:** 1 Department of Industrial Engineering, Universita degli Studi di Padova, 35131 Padova, Italy; hamada.elsayed@unipd.it; 2 Ceramics Department, National Research Centre, 12622 Cairo, Egypt; 3 Department of Civil, Environmental and Architectural Engineering (ICEA) and Inter-Departmental Research Center for the Study of Cement Materials and Hydraulic Binders (CIRCe), University of Padova, 35131 Padova, Italy; michele.secco@unipd.it; 4 Department of Geosciences, University of Padova, 35131 Padova, Italy; federico.zorzi@unipd.it; 5 Institute of Biomaterials, Department of Materials Science and Engineering, University of Erlangen-Nuremberg, 91058 Erlangen, Germany; katharina.ks.schuhladen@fau.de (K.S.); rainer.detsch@fau.de (R.D.); aldo.boccaccini@fau.de (A.R.B.)

**Keywords:** polymer derived ceramics (PDCs), biosilicate ceramics, hardystonite, foams, 3D printed scaffolds, direct ink writing (DIW)

## Abstract

Highly porous bioceramics, based on a complex hardystonite solid solution, were developed from silicone resins and micro-sized oxide fillers fired in air at 950 °C. Besides CaO, SrO, MgO, and ZnO precursors, and the commercial embedded silicone resins, calcium borate was essential in providing the liquid phase upon firing and favouring the formation of an unprecedented hardystonite solid solution, corresponding to the formula (Ca_0.70_Sr_0.30_)_2_(Zn_0.72_Mg_0.15_Si_0.13_) (Si_0.85_B_0.15_)_2_O_7_. Silicone-filler mixtures could be used in the form of thick pastes for direct ink writing of reticulated scaffolds or for direct foaming. The latter shaping option benefited from the use of hydrated calcium borate, which underwent dehydration, with water vapour release, at a low temperature (420 °C). Both scaffolds and foams confirmed the already-obtained phase assemblage, after firing, and exhibited remarkable strength-to-density ratios. Finally, preliminary cell tests excluded any cytotoxicity that could be derived from the formation of a boro-silicate glassy phase.

## 1. Introduction

Silicone polymers, homogeneously mixed with oxide fillers, have been extensively investigated as precursors for silicate bioceramics for the last ten years [1]. A fundamental advantage in all polymer-derived ceramics concerns the possible application of polymer forming techniques, both conventional (e.g., foaming) and advanced (e.g., additive manufacturing technologies), so that a component is first shaped at low temperature and then ‘ceramised’ [2]. Ideally, the ceramic conversion implies just a homogeneous shrinkage, with no microcracking; in actual polymer-derived components, if not thin-walled (e.g., films, fibres, foams) [2], the structural integrity is favoured by the adoption of fillers (either ‘passive’, i.e., simply ‘diluting’ the mass of polymer undergoing transformation, or ‘active’, when fillers react with the matrix and/or with the firing atmosphere) [3,4,5].

In the case of silicates, an additional advantage is the high reactivity of amorphous silica, which results from the ceramic conversion of silicones in air. When mixed with additional oxides, polymer-derived silica leads to highly phase-pure crystalline materials at low temperatures [1]. This reaction obviously occurs once silicone/fillers weight ratios match the expected silica/metal oxide balance of the desired silicates, considering the weight losses in both ceramic conversion of silicones and transformation of fillers (dehydration of hydroxides, decarbonation of carbonates, etc.), operating with fillers of adequate granulometry and phase composition. Nano-sized fillers are generally more reactive and the same oxide, e.g., alumina, may react well in one specific polymorphic form, γ-Al_2_O_3_, instead of another, α-Al_2_O_3_ [1].

Oxide fillers do not simply provide reactants for the amorphous silica matrix. The same transformation of fillers (e.g., dehydration of Mg(OH)_2_ into MgO [6]) may occur below the onset of ceramic conversion of the matrix, leading to some gas release, which, in turn, is exploited for foaming. In other words, fillers may contribute to the chemistry and the shaping of silicate ceramics. In addition, selected fillers (borates, phosphates) [6,7] provide a liquid phase upon firing, enhancing the ionic interdiffusion (diffusion is easier in liquid than in solids) and enabling the development of phase-pure silicates even from coarser fillers. As an example, phase-pure akermanite, Ca_2_MgSi_2_O_7_, was developed from silicone-based mixtures, involving CaCO_3_, as CaO precursors, in both micro- and nano-sized powders, when also including Na-borate [6].

Among different polymer-derived biosilicates, hardystonite (Ca_2_ZnSi_2_O_7_) deserves particular attention. In fact, ceramics based on this phase are currently considered to be very promising alternatives to bioactive glasses in bone tissue engineering applications. They are known to stimulate osteogenic differentiation, cell proliferation and differentiation (among mesenchymal stem cells and bone cells, such as osteoblast-like cells and osteoclasts), and expression of alkaline phosphatase (ALP), osteocalcin, and collagen type I when in contact with human osteoblast (HOB) cells [8,9,10]. Additionally, induction of vascularisation is a crucial part of any successful bone regeneration strategy. In recent years, in vitro studies have shown that bioactive glasses in biomaterial-based tissue engineering (TE) applications are capable of stimulating vascularisation [11,12,13].

Most hardystonite ceramics are derived from the sintering of crystalline powders previously prepared by the sol-gel method [14,15,16]. An alternative is derived from the processing of glass powders, undergoing sintering with concurrent crystallisation [17,18,19]. While the sintering of ceramic powders yields phase-pure materials, the glass approach may yield secondary, inert phases (e.g., Zn aluminate) once the glass formulation does not exactly match that of hardystonite [17].

Previous attempts to create polymer-derived hardystonite were aimed at realising the above-mentioned distinctive coupling of shaping and synthesis, typical of polymer-derived ceramics, with the maximum phase purity [20]. All attempts, however, showed some drawbacks. First, the simplest precursors for CaO and ZnO (CaCO_3_ and ZnO) could not be used for low temperature foaming [20]; second, not strictly thin-walled components, such as 3D scaffolds from direct ink writing of silicone-reactive fillers pastes, were severely microcracked if they did not contain pre-synthesised hardystonite powders as extra, passive filler [21]. An opportunity for low-temperature foaming and some relaxation of stresses developed upon firing (by ceramic conversion and crystallisation of hardystonite) were provided by the adoption of Ca-borate (colemanite) in both hydrated (2CaO 3B_2_O_3_ 5H_2_O) and anhydrous (2CaO 3B_2_O_3_) forms [22].

Besides Ca^2+^ ions, B^3+^ ions, from calcium borate, may also be incorporated in hardystonite-based solid solutions. In fact, hardystonite belongs to the vast group of melilites, i.e., minerals with a distinctive layered structure, offering many possibilities for ionic exchange [22]. Combinations of Zn^2+^ and Si^4+^ ions (in ZnSi_2_O_7_^4−^ sheets, sandwiching Ca^2+^ ions) could be replaced by combinations of Si^4+^ and B^3+^ ions (forming SiB_2_O_7_^4−^ sheets in okayamalite, a B-containing melilite [23]) in tetrahedral coordination. Although successful in providing strong, crack-free foams and scaffolds, the introduction of calcium borate did not lead to phase purity [22]. Additional attempts were dedicated to the inclusion of Sr^2+^ (replacing Ca^2+^) and Mg^2+^ (replacing Mg^2+^) [24]; bioactive ceramics featuring a complex hardystonite solid solution were effectively achieved, but again with limitations on direct processing (glass had to be used as additional filler to enhance the interdiffusion).

The present papers aimed to highlight the conditions for the direct fabrication of highly-porous ceramics from silicone polymers and several oxide fillers, based on a complex hardystonite solid solution, such as (Ca_0.70_Sr_0.30_)_2_(Zn_0.72_Mg_0.15_Si_0.13_) (Si_0.85_B_0.15_)_2_O_7_. This solid solution is interesting, since it couples the hardystonite structure with the simultaneous doping of Sr, Mg, and B ions, known to enhance the functionalities of silicate bioceramics [17,25,26,27,28,29,30]. In particular, the presence of boron is interesting, given its biological activity in terms of osteogenesis and angiogenesis [31]. The obtained ceramics were subjected to a detailed mineralogical analysis as well as to cell tests to assess whether any cytotoxic effect could arise, as an example, from part of the B_2_O_3_ left in the residual glass phase.

## 2. Materials and Methods 

### 2.1. Formulation of Batches for Hardystonite Solid Solutions

The initial reference consisted of 75 mol% hardystonite (Ca_2_ZnSi_2_O_7_) and 25 mol% okayamalite (Ca_2_SiB_2_O_7_), theoretically expressed as Ca_2_Zn_0.75_B_0.5_Si_1.75_O_7_ [22]. We considered the exchange of Ca^2+^ ions (octahedral sites) and Zn^2+^ ions (tetrahedral sites) with Sr^2+^ and Mg^2+^, respectively. The overall chemical formula was Ca_2−x_Sr_x_Zn_0.75−y_Mg_y_B_0.5_Si_1.75_O_7_, with x and y representing the degree of substitution, from 0 up to 30 at%. The batch formulations are reported in Table 1.

The mixtures were calculated according to the silica yield (84 wt%) [1] of the first adopted commercial silicone (MK, Wacker-Chemie GmbH, Munich, Germany). The reactive fillers consisted of commercially available powders, such as CaCO_3_ (<10 μm, Industrie Bitossi, Vinci, Italy), ZnO (<1.48 μm, Sigma Aldrich, Germany), colemanite (Ca_2_B_6_O_11_·5 H_2_O, <1 μm, supplied by CIRCe, University of Padua, Padua, Italy, used after dehydration, by calcination at 500 °C), MgO (30 nm, Inframat Advanced Materials LLC, Manchester, CT, USA), and SrCO_3_ (<10 µm, Bitossi, Italy). 

MK was first dissolved in isopropyl alcohol under magnetic stirring. Clear solutions were then added with the fillers (powders were slowly cast in the MK solutions), again under magnetic stirring, for 15 min. After sonication for 15 min, the mixtures were left to dry at 80 °C overnight in Teflon containers. Finally, dried powders were dry ball milled (Pulverisette 7 planetary ball mill, Fritsch, Idar-Oberstein, Germany), sieved below 90 μm, and pressed in a cylindrical die (diameter of 16.5 mm) at 40 MPa.

A second series of samples, with the same overall formulation (X30Y15, i.e., Ca_1.4_Sr_0.6_Zn_0.64_Mg_0.11_B_0.5_Si_1.75_O_7_), was prepared by replacing MK with H62C silicone (MK, Wacker-Chemie GmbH, Munich, Germany), colloidal silica (SiO_2_, Aerosil R106, Evonik, Germany; 10 wt% of total silica), and quartz sand (<100 μm, Industrie Bitossi, Vinci, Italy). As reported in Table 2, the batches had to be recalculated according to the different silica yield of the silica precursors (58 w% for H62C [1], 100 wt% for colloidal silica and quartz).

All pressed tablets were fired in air at 950 °C, with a 1 h holding time and a heating rate of 2 °C/min, followed by natural cooling.

### 2.2. Direct Ink Writing of Hardystonite Scaffolds

Pastes for direct ink writing experiments were based on MK. As previously done [21], the viscosity of MK-based pastes was adjusted by using colloidal silica, which replaced MK in an amount corresponding to 10 wt% of the total silica content. Colloidal silica as well as the other fillers (CaCO_3_, ZnO, anhydrous colemanite (Ca_2_B_6_O_11_), MgO and SrCO_3_) were all added in an MK paste formed by mixing the silicone with an appropriate solvent (30 vol%). The oxide balance corresponded to X30Y15 and X30Y15B formulations (Table 1). The mixtures were homogenised by ball milling for 5 h at 300 rpm before printing. The printing process was carried out, in air, through a conical nozzle (with a diameter of 0.81 mm—Nordson EFD, Westlake, OH, USA), using a Delta printer (Wasp, Massa Lombarda, Italy). Reticulated structures resulted from the overlapping of filaments of about 0.8 mm diameter, with two different spacing distances (0.8 and 1.6 mm). After printing, the scaffolds were dried in air overnight. The ceramisation process was carried out in air at 950 °C for 1 h, with a heating rate of 0.3 °C/min, followed by natural cooling. During the heating phase, selected samples underwent an intermediate holding step at 590 °C for 3 h.

### 2.3. Preparation of Hardystonite Foams

H62C was used, instead of the MK polymer, as a silica source for foams. The formulations followed those previously shown in Table 1, except for the replacement of MgO with Mg(OH)_2_ (<10 μm, Industrie Bitossi, Vinci, Italy) and the use of colemanite in the hydrated form (Ca_2_B_6_O_11_·5H_2_O). After drying at 60 °C overnight, any H62C-based mixture turned into a viscous paste and was later cast in an aluminium cylindrical moulds (diameter of ~16 mm). The paste was foamed by direct insertion in a furnace set at 420 °C for 10 min. Once extracted from the furnace, hardened foams were separated from the aluminium moulds and subjected to a final thermal treatment. Finally, the ceramisation was performed with the same schedule adopted in the case of the scaffolds.

### 2.4. Characterisations

The bulk density of foams and scaffolds was determined from the weight-to-volume ratio using a calliper and a digital balance. The apparent and true densities of these cellular parts were measured by a gas pycnometer (Helium gas, Micromeritics AccuPyc 1330, Norcross, GA, USA). Morphological details and microstructural characterisations were achieved by optical stereomicroscopy (AxioCam ERc 5s Microscope Camera, Carl Zeiss Microscopy, New York, NY, USA) and SEM (FEI Quanta 200 ESEM, Eindhoven, The Netherlands and JSM JEOL 6490 SEM microscope, JEOL, Tokyo, Japan) equipped with Energy-dispersive X-ray spectroscopy (EDS). 

The compressive strengths of hardystonite (HT)-based foams and scaffolds were measured at room temperature, using an Instron 1121 UTM (Instron, Danvers, MA, USA) operating with a cross-head speed of 0.5 mm/min. Each data point represents the average value of at least 10 individual tests.

The identification of the crystalline phases was performed on finely ground powders by X-ray diffraction (XRD; Bruker AXS D8 Advance, Bruker, Karlsruhe, Germany) with the support of the databases of the crystallographic patterns PDF-2 (ICDD-International Center for Diffraction Data, Newtown Square, PA, USA), the Match! program (Crystal Impact GbR, Bonn, Germany), and the COD database (Crystallography Open Database, www.crystallography.net).

Furthermore, a detailed, multi-analytical approach, comprising microstructural, microchemical, and mineralogical investigations, was applied on pellets the X30Y15 formulation (derived from MK) to quantitatively verify the formation of a stable solid solution phase, including Sr^2+^, Mg^2+^, and B^3+^ ions, in the hardystonite structure. The cross-section of a fired pellet was firstly characterised by SEM-EDS (CamScan MX2500 SEM, Waterbeach, United Kingdom; EDAX, Mahwah, NJ, USA). Subsequently, the same pellet was subjected to a quantitative micro-chemical characterisation by wavelength-dispersive electron microprobe (WDS-EMP; Cameca SX50, CAMECA, Gennevilliers, France). Forty point analyses were acquired from homogeneous areas of the samples, calculating the weight percentages of boron oxide by subtraction from the overall sum of recalculated oxides due to the insensitivity of the instrument to light elements. Then, the mean composition of oxides in the solid solution was determined and used as the input for the calculation of the experimental crystal chemical formula. Finally, quantitative phase analysis (QPA) and structural refinement, based on the Rietveld method [32], were performed on a highly detailed diffraction pattern (14 h data collection with PANalytical X´Pert PRO, PANalytical, The Netherlands), using HighScore Plus 4.7 program package (PANalytical, Almelo, The Netherlands).

Eluates of hardystonite scaffolds were subjected to cytotoxicity assessment tests. Therefore, a suitable bone marrow stromal cell line (ST-2, Deutsche Sammlung für Mikroorganismen und Zellkultur, Germany), isolated from bone marrow of BC8 mice, was used for this purpose. The cells were cultured in CMM (RPMI 1640 medium (Gibco, Germany)) containing 10 vol% fetal bovine serum (FBS) (Sigma-Aldrich, Germany), 1 vol% penicillin/streptomycin (Sigma-Aldrich), and 1 vol% Glutamax (Gibco)). For these tests, 100,000 ST2 cells were seeded in 1 mL CCM in 24-well plates for 24 h (incubated at 37 °C in a humidified atmosphere of 95% air and 5% CO_2_). Simultaneously, scaffold samples were incubated in CCM for 24 h in a ratio 5 g:5 mL. CCM was removed from the samples and samples were then diluted with CCM to form 1%, 0.1%, and 0.01% dilutions. Finally, these dilutions were used to cultivate the cells for 48 h (with pure CCM as control). For assessing the influence of different material supernatant concentrations on the viability of the cultivated cells, a WST-8 assay (Sigma-Aldrich) was used. The amount of released VEGF from ST-2 cells into the cell culture medium was measured by using a RayBio Human VEGF ELISA (Enzyme-Linked Immunosorbent Assay) kit. In order to observe the morphology of the bone marrow stromal cells cultivated with different dilutions of the of hardystonite scaffolds, H&E (Hematoxylin & Eosin) staining was performed.

## 3. Results

### 3.1. Phase Evolution

The starting point for the present investigation consisted of the theoretical solid solution between Zn- and B-based melilites, such as hardystonite (HSt, Ca_2_ZnSi_2_O_7_) and okayamalite (Ok, Ca_2_B_2_SiO_7_), as previously studied [22]. Silicone-filler mixtures designed for a stoichiometry 75 mol% HSt–25 mol% Ok (Ca_2_Zn_0.75_B_0.5_Si_1.75_O_7_) did not yield a single phase, forming a solid solution with approximate stoichiometry of Ca_2_Zn_0.83_B_0.33_Si_1.83_O_7_, accompanied by traces of wollastonite (CaSiO_3_) and calcium borate [22]. However, the formulation had significant advantages in producing hardystonite in a single process (no treatment of a previously synthesised powder) at a particularly low temperature (<1000 °C).

**Figure 1:**
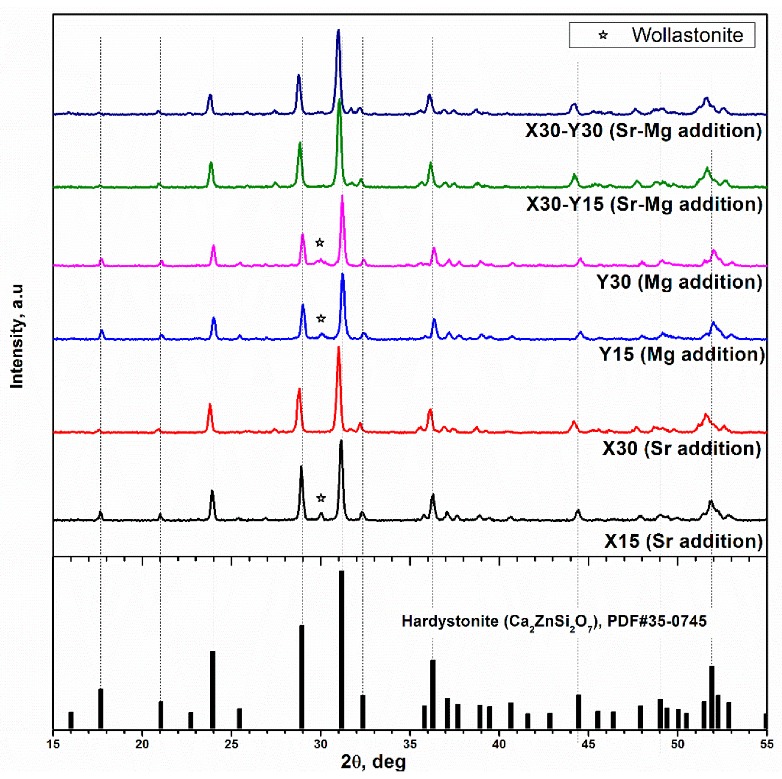
XRD analysis different of hardystonite solid solutions compared with reference pattern for pure hardystonite.

The ceramic from formulation X15 confirmed the feasibility of a single process production at low temperature, but it evidently did not follow the ideal structure (single phase, consisting of Ca_1.7_Sr_0.3_Zn_0.75_B_0.5_Si_1.75_O_7_), as seen in the diffraction patterns in Figure 1. Most diffraction peaks matched the ones of pure hardystonite (PDF#35-0745), except for a small downshift in the 2*θ* positions. This downshift is reasonable, owing to the Sr doping; Sr hardystonite (Sr_2_ZrSi_2_O_7_) is known to feature a diffraction pattern similar to that of hardystonite, but with all peaks shifted at lower 2*θ* positions [26]). The peak at 2*θ*~30° could be attributed, as in previous studies, to wollastonite (CaSiO_3_, PDF#10-0489). 

Secondary phases were likely reduced in the X30 ceramic (stoichiometry Ca_1.4_Sr_0.6_Zn_0.75_B_0.5_Si_1.75_O_7_) since all diffraction peaks could be ascribed to hardystonite solid solution; the increased downshift in the 2*θ* positions was consistent with the enhanced incorporation of extra ions. 

For the formulations Y15 and Y30 (corresponding to the replacement of 15% and 30% of Zn^2+^ ions, respectively), the assessment of the incorporation effect of Mg^2+^ from shifts in the 2*θ* positions was difficult since the pattern of magnesium-based melilite, akermanite (Ca_2_MgSi_2_O_7_, PDF#79-2425), is nearly indistinguishable from that of hardystonite. Again, a secondary phase was evident.

X30Y15 and X30Y30 ceramics, corresponding to the simultaneous inclusion of Sr^2+^, Mg^2+^, and B^3+^ ions in the hardystonite structure, confirmed the reduction of secondary phases found with X30. Strontium ions likely had a ‘triggering’ action. That is, the distortion in the spacing between zinc silicate layers, caused by the larger Sr^2+^ ions compared to the Ca^2+^ ions, evidently balanced the distortions in the same layers occurring upon replacement of Zn^2+^ and Si^4+^ ions with Mg^2+^ and B^3+^ ions.

The successful formation of a solid solution with such a high degree of complexity (six oxides simultaneously present) is promising since it could be the basis for the incorporation of a number of dopants. In other words, hardystonite solid solutions could represent ‘hosts’ for many ions, each with specific functions in tissue engineering, thereby offering an alternative to 45S5 bioactive glass (the doping of 45S5 is one of the most promising topics in the research on biomaterials) [29,33,34]. 

**Figure 2:**
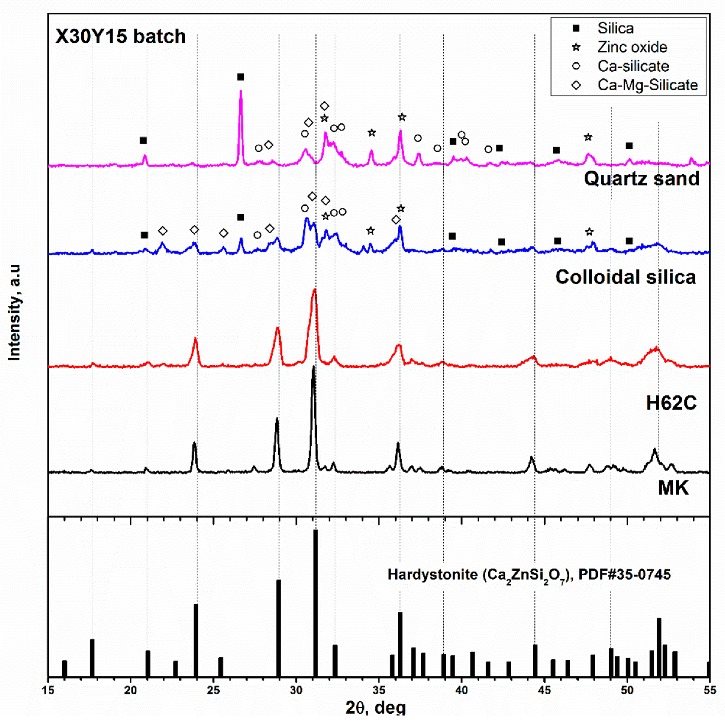
Assessment of reactivity of different silica sources in the development of hardystonite solid solution.

Considering the Sr and Mg amounts in the already recognised hardystonite-based biomaterials [26,27], we selected the X30Y15 formulation for additional experiments, aimed at evidencing (for the first time) the specific role of preceramic polymers. In fact, silicones, as a silica source, had a synergistic effect with oxide fillers on phase evolution. Figure 2 shows the diffraction patterns of ceramics corresponding to the X30Y15 formulation obtained (according to the same firing schedule) from two different silicones and two different forms of silica. One could suppose that using calcium borate which leads to a liquid phase, could provide a strong ‘fluxing’ action and dissolve most parts of the other components at the early stages of firing, forming a viscous mass and later crystallising into a hardystonite solid solution. It was evident that neither quartz sand nor the more reactive colloidal silica led to the expected phase. Calcium and calcium-magnesium silicates (Ca_2_SiO_4_, PDF#31-0298, and CaMgSi_2_O_6_, PDF#89-1484) could be found along with quartz (PDF#86-1560) and ZnO (PDF#89-0511).

Amorphous silica from the oxidation of silicone had a confirmed high reactivity towards oxide fillers. Switching from MK to H62C did not modify the developed phase given the position and the intensity of diffraction peaks; H62C likely resulted in just a reduction in crystal size, considering the broadening of diffraction peaks, as seen in Figure 2.

**Figure 3:**
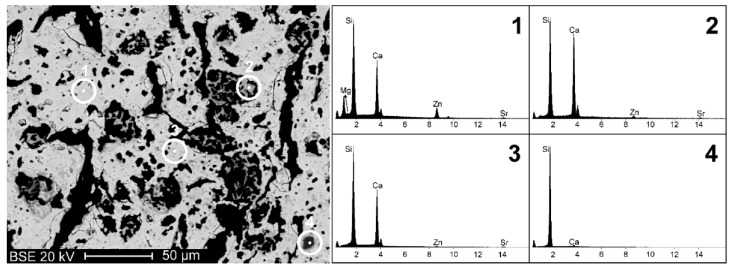
Backscattered electron image (BEI) of sample X30Y15 and EDS analyses on a portion of solid solution phase (spectrum **1**) and on portions of accessory phases (spectra **2**, **3** and **4**).

The pellets from MK were subjected to a refined microstructural and mineralogical analysis. SEM-EDS analyses (Figure 3) showed that the X30Y15 sample constituted a fairly homogeneous porous matrix with a dominating content of Ca and Si, but also included Zn, Sr, and Mg (spectrum 1 in Figure 3). Such experimental evidence indicates a successful incorporation of the elements in the solid solution (B was not detectable by EDS analysis). However, some portions with different chemical composition, i.e., with a darker colour in the backscattered electron image, were detected. The Ca/Si ratio in these portions presented significant variations, passing from high levels (spectra 2–3), typically presented by wollastonite (CaSiO_3_, i.e., Ca/Si = 1), to particularly low levels, corresponding to almost pure silica (spectrum 4). Such experimental data indicate that the reaction process still did not lead to a full incorporation of all the chemical constituents into a single crystalline phase; however, hardystonite solid solution was largely dominant.

**Figure 4:**
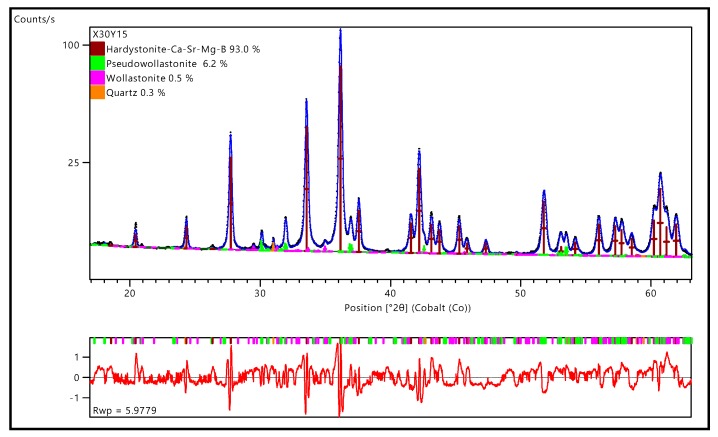
Rietveld refinement of X30Y15.

The mean oxide composition of the hardystonite solid solution, reported in Table 3, may be summarised by the crystal chemical formula of (Ca_0.70_Sr_0.30_)_2_(Zn_0.72_Mg_0.15_Si_0.13_) (Si_0.85_B_0.15_)_2_O_7_. This formula was confirmed by the Rietveld refinements, illustrated in Figure 4, performed on a high-resolution diffraction pattern (14 h data collection, Co anode instead of Cu anode used for preliminary phase identification studies).

The chemical formula of hardystonite from EMP-WSD analysis was implemented in a melilite structural model Panalytical ICSD database (akermanite 280405). Atomic positions and occupancies were refined (see Table 4) with the exception of the 2a Wyckoff position, including 3 atomic species (Si, Zn, Mg). The Rietveld refinement led to an excellent fitting of experimental data and evidenced secondary phases, in the forms of pseudo-wollastonite, wollastonite 2M, and quartz, in limited amounts (not exceeding 7 wt%). The presence of pseudo-wollastonite was also confirmed by the characteristic cathodo-luminescence [35] observed during EMP-WDS analysis. 

### 3.2. Obtainment of Scaffolds and Foams

**Figure 5:**
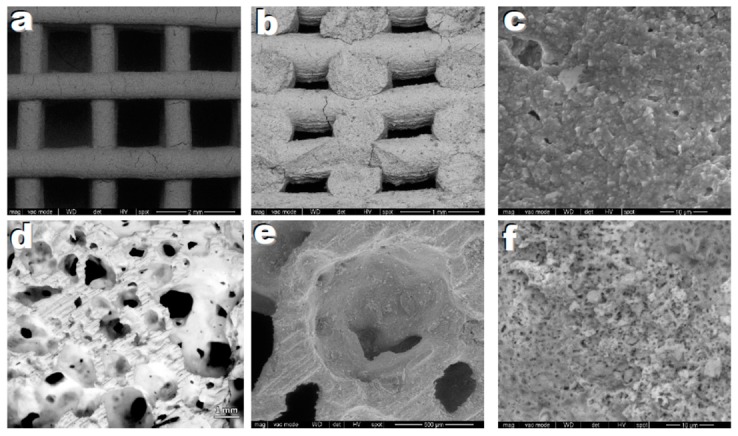
Microstructural details of highly porous ceramics based on hardystonite solid solution, after firing at 950 °C; (**a**–**c**) printed scaffolds; (**d**–**f**) Foams.

Scaffolds and foams were prepared starting from MK- and H62C-based mixtures, respectively. Figure 5 shows selected images from scaffolds based on the X30Y15 formulation from direct ink writing of silicone pastes. Owing to the use of anhydrous borate, the residual porosity was quite limited; however, some microcracks were still visible (Figure 5a,b). These cracks are ascribable to the gas released from fillers and from the ceramic transformation of the MK silicone (still representing the dominant silica source). The filaments, in any case, showed a good interpenetration (Figure 5b); high magnification details (Figure 5c) showed the formation of a multitude of tiny crystals. The scaffolds, in terms of strength (Table 3) for both settings of filament spacing (800 and 1600 µm), compared favourably with other hardystonite scaffolds with similar porosity [36].

The water vapour released from within H62C still in its polymer state, from the use of hydrated fillers (hydrated Ca borate and Mg(OH)_2_), led to a very remarkable foaming at 420 °C. The ceramic transformation confirmed an abundant porosity (>80 vol%), completely open and with wide interconnections (>100 µm), reflecting the main requirements of a scaffold for tissue engineering applications [37], as shown in Figure 5d. The high magnification details illustrate the presence of a substantial porosity in the cell walls (Figure 5e,f), resulting from the gas released upon ceramic conversion, as well as the macro-porosity determined by the low temperature foaming, caused by dehydration of some fillers. Porous walls are known to promote cell adhesion, absorption of metabolites, and faster controlled rates for the release of ionic dissolution products [38]. The observed crushing strength (Table 5) is in the order of that of silicate foams with the same porosity [14,39].

### 3.3. Preliminary Cell Tests

The phase assemblage, resulting from the mineralogical analysis, supports the hypothesis of a crystallinity degree not exceeding 80 wt%, as seen in the relatively high content of MgO detected in the hardystonite solid solution (corresponding to 93% of the crystals), compared to the reference stoichiometry (X30Y15). This estimation is consistent with the assumption of MgO completely embedded in the hardystonite solution and the other oxides being distributed, except for minor crystal phases, in a glass phase surrounding the silicate crystals. This implies, above all, that the concentration of B_2_O_3_ (in an amount of about 30 wt% of the glassy phase) could be critical for the application of the developed porous materials in tissue engineering. In fact, although present in many bioceramics [29,30], B_2_O_3_ is quite controversial because of the potential toxicity of boron released in solution as borate ions (BO_3_)^3−^ [40,41].

**Figure 6:**
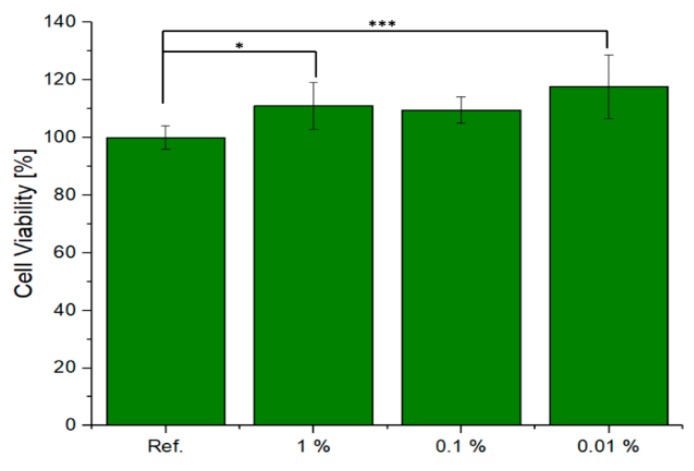
Relative viability of ST2 cells cultured with IDPs of tested material, One-way ANOVA statistical analysis denotes significant difference (*** *p* < 0.001; * *p* < 0.05).

Preliminary cell tests were performed on X30Y15 scaffolds with the specific purpose of elucidating any cytotoxic effect. The cell viability of ST2 cells in the presence of the scaffold eluates is shown in Figure 6. The reference, which relates to cells cultured only with CCM, was normalised to 100%. Quantitative assessment after 48 h of culture shows that there was an increase in cell viability with a decrease in the concentration of the scaffold eluates in the cell culture medium. In the case of 0.01% dilution, the highest cell viability was achieved by the 0.1% concentration. The values are statistically relevant as the difference was greater than *p* = 0.001. The scaffold eluates were found to not be cytotoxic after 48 h of incubation in contact with bone marrow stromal cells.

**Figure 7:**
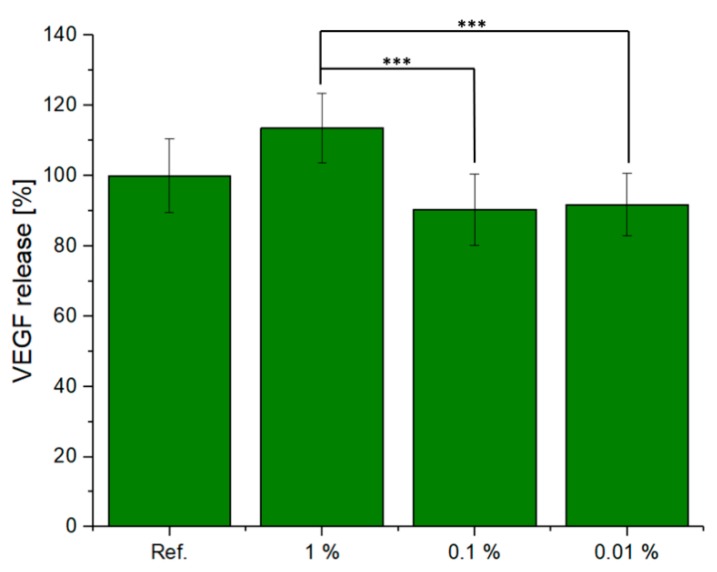
VEGF release from ST2 cells treated with different dilutions of IDPs of the tested material, one-way ANOVA statistical analysis denotes significant difference (*** *p* < 0.001).

In Figure 7, the VEGF released from the ST2 cells cultured in CCM with different dilutions (1%, 0.1%, and 0.01%) of scaffold is shown. Measuring the increase in VEGF concentration when cells are exposed to dissolution products of biomaterials is a suitable in vitro methodology [42] to assess the potential angiogenic effect of the material being investigated, given that increased VEGF will attract endothelial cells, being thus a marker for the vascularisation potential of the biomaterial. The eluates of this scaffold increased VEGF secretion with increasing supernatant concentration. The 1% supernatant sample showed the highest release of VEGF for all three dilutions. These results were also in accordance with the data obtained from the cell viability study. However, the results also indicated no significant variation as a function of the reference sample.

**Figure 8:**
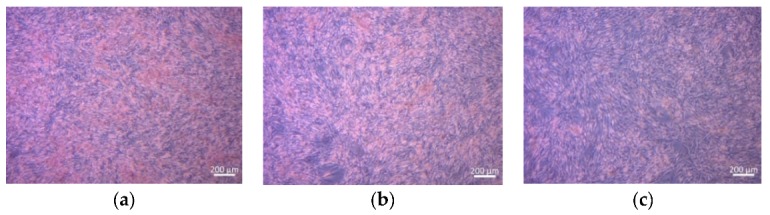
Light microscopy images of H&E-stained ST2 cells cultured with IDPs in CCM with different dilutions ((**a**) = 1%, (**b**) = 0.1%, and (**c**) = 0.01%) of tested material.

Representative light microscope images of bone marrow stromal ST-2 cells (H&E stained), treated with the supernatant of the scaffold at different concentrations (0.01–1 of scaffold) after 48 h of incubation, are shown in Figure 8. The images demonstrate clearly that ST-2 cells grew in contact with the supernatant without any appreciable change in cell morphology. A dense formed cell layer in contact with all sample supernatants was observed. 

## 4. Conclusions

Although still not phase pure, porous ceramics based on a complex hardystonite solid solution were successfully manufactured by the direct firing of silicone-based mixtures at only 950 °C in air. The reactivity of amorphous silica provided by silicones was much higher than that of more conventional silica sources when combined with the same additives (precursors of the other oxides). The production of the hardystonite solid solution involved the unprecedented combination of six oxides, with Mg^2+^ and B^3+^ ions modifying the ZnSi_2_O_7_^4−^ sheets, and Sr^2+^ ions partially replacing the Ca^2+^ ions sandwiched between the same sheets.

The use of silicones mixed with fillers, besides favouring synthesis, enabled the application of simple shaping technologies, such as direct ink writing and direct foaming, at low temperature, with silicones still in the polymer state. All developed porous hardystonite ceramics compared well, in terms of strength-to-density ratio, with the analogous materials presented in the literature. The direct foaming, in particular, is promising for the possibility of obtaining cellular bodies with both well-interconnected macropores and porous cell walls. According to the results of preliminary cell tests, the developed ceramics were not cytotoxic.

## Figures and Tables

**Table 1:** Formulations adopted with relative number of substituted ions and chemical formula.

Sample Type	Chemical Formula	Batch Formulation for 100 g Ceramic Yield					
		MK Silicone (g)	CaCO_3_ (g)	ZnO (g)	Colemanite (Ca_2_B_6_O_11_) (g)	SrCO_3_ (g)	MgO (g)
X15	Ca_1.7_Sr_0.3_Zn_0.75_B_0.5_Si_1.75_O_7_	42.3	51.93	20.6	9.1	14.99	0
X30	Ca_1.4_Sr_0.6_Zn_0.75_B_0.5_Si_1.75_O_7_	42.3	41.76	20.6	9.1	29.99	0
Y15	Ca_2_Zn_0.64_Mg_0.11_B_0.5_Si_1.75_O_7_	42.3	62.1	17.51	9.1	0	1.53
Y30	Ca_2_Zn_0.53_Mg_0.22_B_0.5_Si_1.75_O_7_	42.3	62.1	14.42	9.1	0	3.06
X30Y15	Ca_1.4_Sr_0.6_Zn_0.64_Mg_0.11_B_0.5_Si_1.75_O_7_	42.3	41.76	17.52	9.1	29.99	1.53
X30Y30	Ca_1.4_Sr_0.6_Zn_0.53_Mg_0.22_B_0.5_Si_1.75_O_7_	42.3	41.76	14.42	9.1	29.99	3.06

**Table 2:** Alternative batches for X30Y15 hardystonite ceramics.

Silica Precursor Type	Batch Formulation for 100 g Ceramic Yield					
	Silica Precursor (g)	CaCO_3_ (g)	ZnO (g)	Colemanite (g)	SrCO_3_ (g)	MgO (g)
H62C	63.4	41.76	17.52	9.1	29.99	1.53
Colloidal silica	35.5	41.76	17.52	9.1	29.99	1.53
Quartz sand	35.5	41.76	17.52	9.1	29.99	1.53

**Table 3:** Mean chemical composition, expressed in oxides wt%, of the hardystonite solid solution constituting the sample X30Y15, as determined by EMP-WDS.

Oxide	SiO_2_	CaO	SrO	ZnO	MgO	B_2_O_3_
wt%	34.07 ± 0.21	24.00 ± 0.34	18.82 ± 0.44	17.98 ± 0.20	1.88 ± 0.06	3.24 ± 0.24

**Table 4:** Detail of atomic positions and occupancies of the main crystal phase.

ATOM	WYCKOFF	S.O.F.	X	Y	Z	Biso
O1	8f	1.000000	0.3145(2)	0.5777(1)	0.2073(2)	0.500000
O2	4e	1.000000	0.1401(2)	0.6401(2)	0.7461(2)	0.500000
O3	2c	1.000000	0.000000	0.500000	0.1607(4)	0.500000
Si1	2a	0.130000	0.000000	0.000000	0.000000	0.500000
Zn	2a	0.718765	0.000000	0.000000	0.000000	0.500000
Mg	2a	0.150000	0.000000	0.000000	0.000000	0.500000
Sr	4e	0.2995(8)	0.666(8)	0.166(8)	0.4943(2)	0.500000
Ca	4e	0.7005(8)	0.666(8)	0.166(8)	0.494200	0.500000
B	8f	0.150(2)	0.141(3)	0.639(3)	0.0592(1)	0.500000
Si2	8f	0.850(2)	0.141(3)	0.639(3)	0.0592(1)	0.500000

**Table 5:** Physical and mechanical properties of cellular Sr/Mg-doped hardystonite ceramics.

Sample Type	-	Bulk Density (g/cm^3^)	Total Porosity (vol%)	Open Porosity (vol%)	Compressive Strength (MPa)
Scaffolds	800 *	1.38 ± 0.01	56 ± 2	56 ± 2	4.6 ± 0.5
	1600 *	0.90 ± 0.05	71 ± 1	71 ± 1	1.6 ± 0.2
Foams	-	0.60 ± 0.02	82 ± 1	82 ± 1	1.5 ± 0.2

* Spacing between filaments (µm).
